# Supplementary material for: Isolation and Characterisation of Alongshan Virus in Russia
Source: Viruses. 2020 Mar 26;12(4):362. doi: 10.3390/v12040362 (PMC7232203; doi:10.3390/v12040362)
Supplement: Supplementary file 1 [file viruses-12-00362-s001.pdf]

Table S1. Specific primers for amplification of genome segments 1 and 2 of Alongshan virus and IRE/CTVM19-associated rhabdovirus.

Name of primers	Nucleotide sequence	Primer direction	Genome locus	Amplicon size, bp	Temperature, ^{0}C
MiassF	GGTACACGGACCTGGG ATCCTATTG	Forward	segment 1	825	50
MiassR	TCTCTGACTCCTGTTCT AATC	Reverse	segment 1		
JMun1S	TTAAAARCGGCCAGCC TTNRYTGCAAGTGCA	Forward	segment 2	1800	55
Miass_gly_1R	ACCAGGTTGGTCAAGG CAAT	Reverse	segment 2		
Miass_gly_3F	TGGATCAGCTCACACC ACAC	Forward	segment 2	333	53
Miass_gly_3R	TCACCGTCACAGTGGA ATGG	Reverse	segment 2		
JVsenseV1add	GGTACACGGACCTGGG ATCCTATTG	Forward	segment 1	275	50
JVasenseV1	TAGGCCCTGACCAGCC ACGCTCC	Reverse	segment 1		
JVsenseV1add	GGTACACGGACCTGGG ATCCTATTG	Forward	segment 1	819	50
Mi1-2320as	TCTCTGACTCCTGTTCT AATC	Reverse	segment 1		
Rhabdo _L_1F	GGGTTTGTGGTTAATTT GTC	Forward		392	50
Rhabdo_L_1R	AGTGAGGACTGGATAA AAGA	Reverse			

Table S2. List of Jingmenvirus group sequences used for the discovery of the conservative elements within segment 2.

Accession number	Virus name (according to GenBank)	Used for analysis
MH158416	Alongshan virus strain H3	ALSV [1]
MN107154	Alongshan virus strain Kuutsalo-23	ALSV
MN107158	Alongshan virus strain Haapasaari-18	ALSV
MN095520	Jingmen tick virus isolate JMTV/I.ricinus/France	ALSV
MH688530	Yanggou tick virus strain YG	Yanggou virus [2]
MH688533	Yanggou tick virus strain 16-T2	Yanggou virus

MH688537	Yanggou tick virus strain 17-L1	Yanggou virus
KJ001580	Jingmen Tick Virus isolate SY84	JMTV[3]
KY523073	Mogiana tick virus isolate MGTV/V4/11	JMTV
MG703254	Amblyomma virus GXTV108	JMTV
MH133315	Jingmen tick virus isolate Kosovo 2013-17-266	JMTV
MH133319	Jingmen tick virus isolate Kosovo 2014-C-K14-1C	JMTV
MH133323	Jingmen tick virus isolate Kosovo 2015-A-K15-1A	JMTV
MH155890	Jingmen tick virus isolate JTMV_1	JMTV
MH155894	Jingmen tick virus isolate JTMV_3	JMTV
MH155905	Jingmen tick virus isolate JTMV_100	JMTV
MH814978	Rhipicephalus associated flavi-like virus isolate YNTV4	JMTV
MK174244	Jingmen tick virus isolate XJ58	JMTV
MK174245	Jingmen tick virus isolate XJ61	JMTV
MK174246	Jingmen tick virus isolate XJ77	JMTV
MK174247	Jingmen tick virus isolate XJ155	JMTV
MK174248	Jingmen tick virus isolate XJ335	JMTV
MK174249	Jingmen tick virus isolate XJ363	JMTV
MK673134	Kindia tick virus isolate KITV/2017/1	JMTV
MN025513	Jingmen tick virus isolate TTP-Pool-3b	JMTV
MN025517	Jingmen tick virus isolate TTP-Pool-19	JMTV
MN095524	Jingmen tick virus isolate JMTV/Rh.microplus/Am.variegatum/French Antilles	JMTV
MN095528	Jingmen tick virus isolate JMTV/Am.testudinarium/Lao PDR	JMTV
MN486259	Jingmen tick virus isolate T36	JMTV
MN486258	Jingmen tick virus isolate T17	JMTV
MN486257	Jingmen tick virus isolate T15	JMTV
MN486256	Jingmen tick virus isolate T14	JMTV
MN095532	Jingmen tick virus isolate JMTV/Pteropus lylei/Cambodia	None[4]
KX377514	Jingmen tick virus strain RC27	None

[1] – VP1a ORF was used in the codon alignment of Alongshan viruses (ALSV)

[2] – VP1a ORF was used in the codon alignment of Yanggou viruses

[3] – VP1 ORF was used in the alignment of Jingmen tick virus (JMTV)

[4] – sequence was not used in any of the alignments above due to large number of unidentified nucleotides. nuORF was confirmed to be intact.

Table S3 Additional Alongshan virus isolates from Chelyabinsk region detected by screening of tick pools using a heminested RT-PCR for the flavivirus NS5 gene.

Strain	Tick species	Year, region (GPS)	GenBank access. no.
Miass501	*I. persulcatus*	2014, Chelyabinsk region, Ilmen State Reserve (55°01.287′N 060°10.097′E)	MT210222
Miass502	*I. persulcatus*		MT210220
Miass506	*I. persulcatus*		MT210219
Miass508	*I. persulcatus*		MT210221
Miass510	*I. persulcatus*		MT210225
Miass515	*I. persulcatus*		MT210223
Miass523	*I. persulcatus*		MT210224

Figure S1. Phylogenetic tree of all full segment 2 sequences of the Jingmenvirus group.

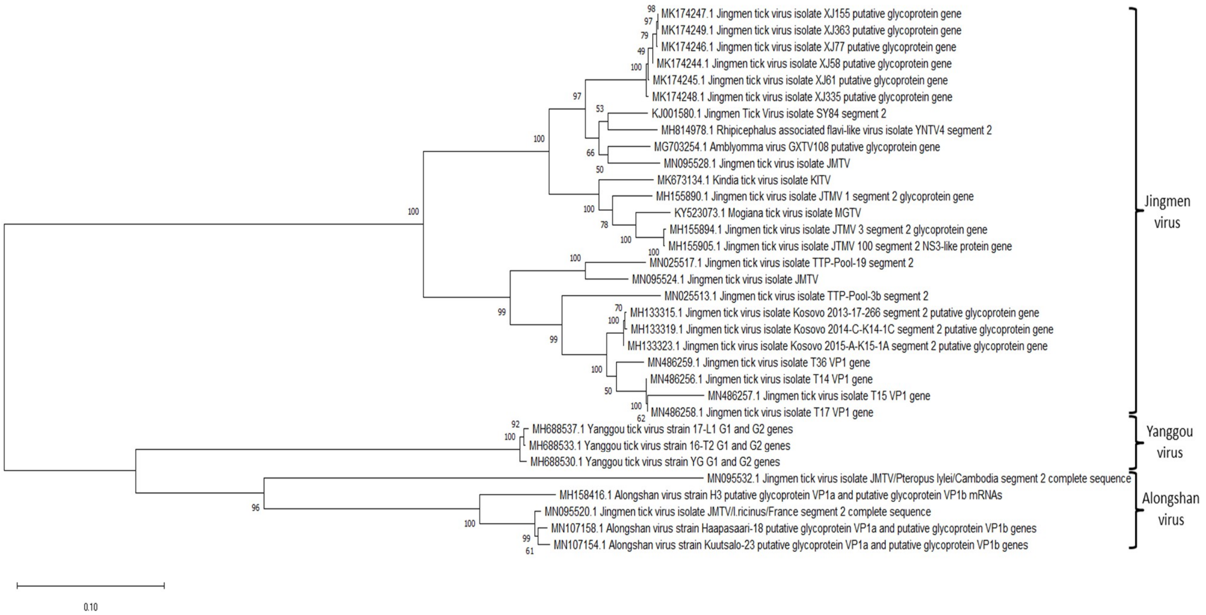

The evolutionary history was inferred by using the Maximum Likelihood method and Tamura-Nei model [1]. The tree with the highest log likelihood (-20097.90) is shown. The percentage of trees in which the associated taxa clustered together is shown next to the branches. Initial tree(s) for the heuristic search were obtained automatically by applying Neighbor-Join and BioNJ algorithms to a matrix of pairwise distances estimated using the Maximum Composite Likelihood (MCL) approach, and then selecting the topology with superior log likelihood value. The tree is drawn to scale, with branch lengths measured in the number of substitutions per site. This analysis involved 33 nucleotide sequences. There were a total of 2282 positions in the final dataset. Evolutionary analyses were conducted in MEGA X [2]

Figure S2. Results of the RT-PCR at selected time points of Alongshan virus strain Miass527 persistence in the IRE/CTVM19 tick cell line.

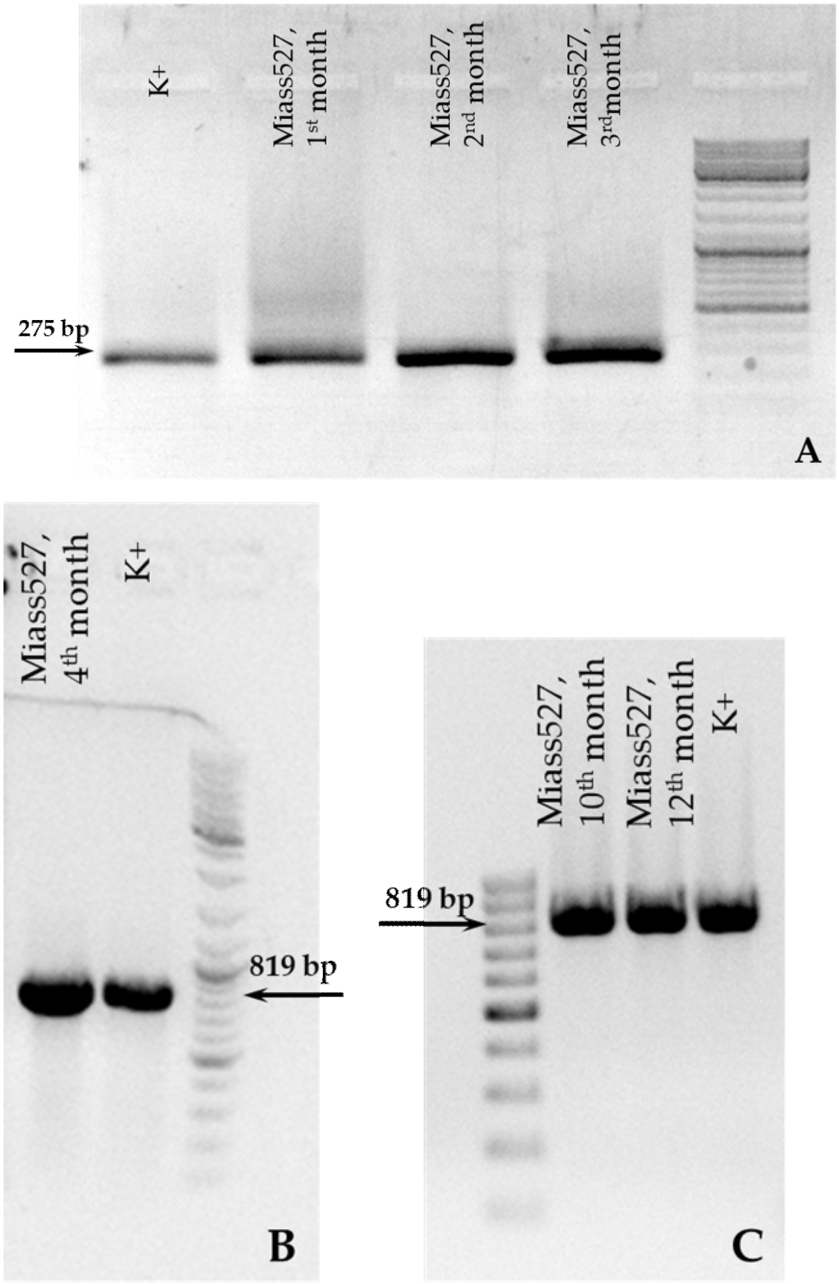

A – Culture supernate of IRE/CTVM19 infected with strain Miass527 for 1, 2 and 3 months; primers JVsenseV1add – JVasenseV1

B – Culture supernate of IRE/CTVM19 infected with strain Miass527 for 4 months; primers JVsenseV1add – Mi1-2320as

C – Culture supernate of IRE/CTVM19 infected with strain Miass527 for 10 and 12 months; primers JVsenseV1add – Mi1-2320as

K+ - positive control

Figure S3. The range of virion size detected in transmission electron microscopy of strain Miass527 of Alongshan virus.

Most of the virions were spherical particles with a diameter of 40.5 ± 3.7 nm.

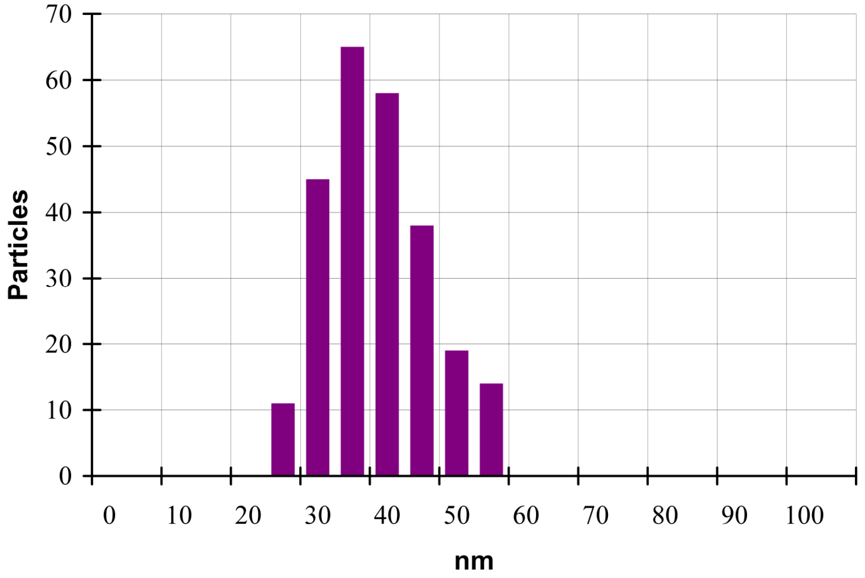

Figure S4. The size of the small spherical particles detected in transmission electron microscopy of strain Miass527 of Alongshan virus.

The small spherical particles had a diameter of 13.1 ± 2.1 nm.

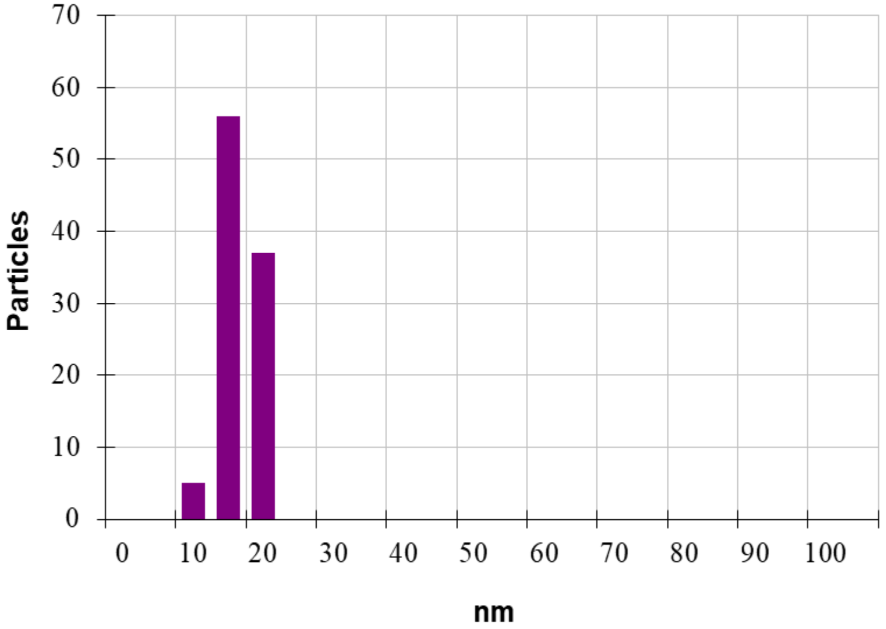

Figure S5. The size of the small spherical particles detected in transmission electron microscopy of ultracentrifuged supernate from uninfected IRE/CTVM19 cells.

The small spherical particles had a diameter of 15.7 ± 1.76 nm.

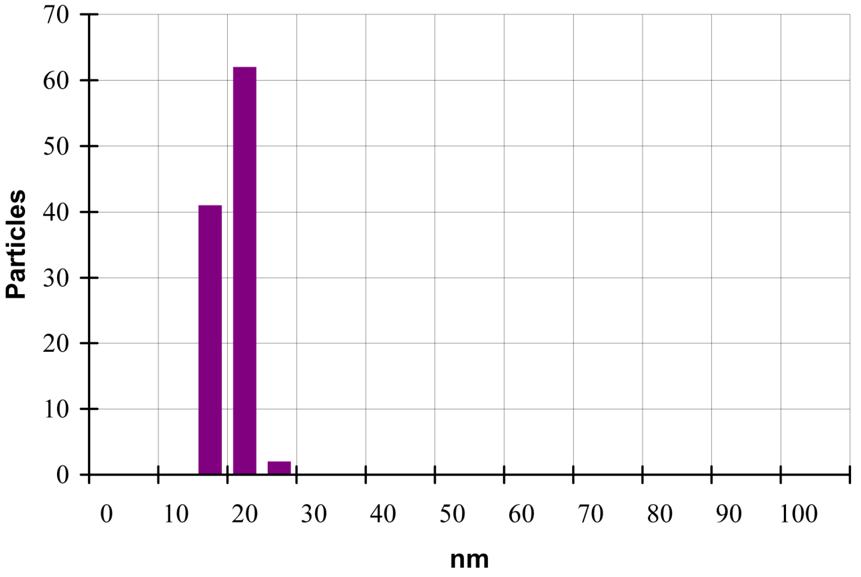

Figure S6. Predicted RNA structure downstream of the frameshift region in segment 4 of Alongshan viruses (ALSV).

The frameshift site is colored teal (turquoise). Red, green and grey coloring represents paired groups of nucleotides in the structure that may cause frameshifts. Structure was predicted with the pAliKiss algorithm [3] using alignment of the 201 nt region of ALSV strains H3, Miass527, Miass519, Kuutsalo-23 and Haapasaari-18 as an entry.

1
gguuuuucaguaggggaggggaauacccagccaccccccucccgaaccgacaggaagccugucggagaagggaag
agcuggauaccgaacugggcugaagugagcagggacuacuugguuagacucggaaagaaggccgcacucauggg
agagggcuacgacaucauccuugaugaggcuagggauuuguucccaaccucc 201
(-75.94 = -74.90 + -1.04)
.........[[..{{{{{{{{.]]..<<<<<....}}}}}}}}...((((((((...))))))))...........>>>>>...[[[[....{{{{....(((.((((.....)))).)))...]
]]]..<<<.((((...(((....)))..))))....(((((....))))).}}}}.((((.....))))....>>>

Figure S7. Predicted RNA structure downstream of the frameshift region in segment 2 of Alongshan viruses (ALSV).

The frameshift site is colored teal (turquoise). Red, green, grey, purple and blue coloring represent paired groups of nucleotides in the structure that may cause frameshifts. Structure was predicted with the pAliKiss algorithm [3] using alignment of the 201 nt region (downstream to the proposed frameshift site) of ALSV strains H3, Miass527, Miass519, Kuutsalo-23 and Haapasaari-18 as an entry.

1
aaaaaacgucaucagaccguccaugaggcuuuuaccgauuacacccccgugccaaagcauugguacgacuggcuu
ucgagcugguuuggccacaucacgggagguaucgagagggcuuaucggaucaucucaugggcggucgaguuu
guuacuagcaacgcuccaacugugcuuguggucaucaucauggccacaccccuc 201
(-75.50 = -74.66 + -0.84)
.......[[[....{{{{{{{{{{{{]]]......((((...(((((((((.........((((.(((((((((....)))))).)))))))...)))))).))))))).<<<<<............
..}}}}}}}}}}}}(((((((((...))))).)))).........(((((((((....))))))))).>>>>>

Figure S8. Synonymous site conservation analysis of the Yanggou virus VP1a ORF.

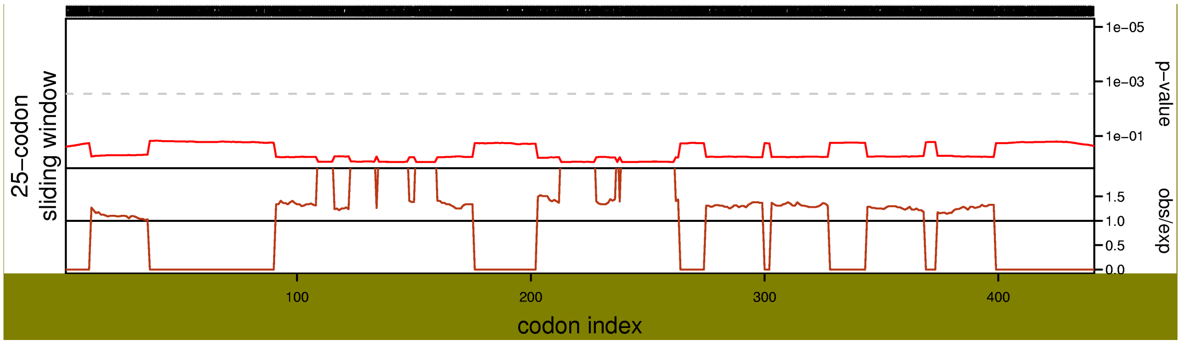

The top panel depicts the probability that the degree of ORF conservation within a 25-codon sliding window could be obtained under neutral evolution. The grey dashed line indicates p=0.005 significance (after correcting for multiple tests, where the number of tests is the length of a coding sequence divided by the window size). The bottom panel displays the relative amount of synonymous-site conservation at a 25-codon sliding window by showing the ratio of the observed number of synonymous substitutions to the expected number.

The analysis was done using Synplot2 program [4].

References

1. Tamura, K.; Nei, M. Estimation of the number of nucleotide substitutions in the control region of mitochondrial DNA in humans and chimpanzees. *Mol. Biol. Evol.*

1993, *10*.
2. Kumar, S.; Stecher, G.; Li, M.; Knyaz, C.; Tamura, K. MEGA X: Molecular evolutionary genetics analysis across computing platforms. *Mol. Biol. Evol.* **2018**, *35*, 1547–1549.
3. Janssen, S.; Giegerich, R. The RNA shapes studio. *Bioinformatics* **2015**, *31*, 423–425.
4. Firth, A.E. Mapping overlapping functional elements embedded within the protein-coding regions of RNA viruses. *Nucleic Acids Res.* **2014**, *42*, 12425–12439.
